# The Standard Dictionary of Funk & Wagnalls

**Published:** 1894-12

**Authors:** 


### The Standard Dictionary of Funk & Wagnalls.

It is remarkable to note the high testimonials the Standard Dictionary continues to receive from leading educational authorities in Europe and America, as Dr. John T. Duffield, of Princeton College, N. J., who writes: "It will be conspicuous among the enduring monuments of intellectual life at the close of the nineteenth century. * * * For comprehensiveness of vocabulary, accuracy of definition, judicious arrangement of material, instructive illustrations and admirable typography, it is superior to any other work of its class, and ere long will supersede them, and be recognized as the Standard Dictionary."

*Nature*, London, Eng., J. Norman Lockyer, the celebrated astronomer, editor, says: "It passes the wit of man to suggest anything which ought to have been done that has not been done to make the dictionary a success."

